# Facilitating the action of community representatives in a health service: the role of a community participation coordinator

**DOI:** 10.1186/1472-6963-13-154

**Published:** 2013-04-29

**Authors:** Sally Nathan, Jeffrey Braithwaite, Niamh Stephenson

**Affiliations:** 1 School of Public Health and Community Medicine, UNSW, Sydney, NSW, 2052, Australia; 2 Australian Institute of Health Innovation, Centre for Clinical Governance Research, UNSW Medicine, UNSW, Sydney, NSW, 2052, Australia

## Abstract

**Background:**

Commitments to community participation are common in health policy, yet ways to maximise the input and impact of community representatives in health service delivery and care remain elusive, lack empirical evidence and are under-theorised.

**Methods:**

The role of Community Participation (CP) Coordinators involved in an Australian health service were examined in a triangulated multi-method, multi-site ethnographically informed three year study. Formal fieldwork involved observation of just over 42 hours of meetings together with informal interactions in the field with staff and community members and in-depth interviews and discussions with 10 Community Representatives, 19 staff and the seven CP Coordinators employed during the study period.

**Results:**

Four key roles that Community Participation Coordinators undertake to support and facilitate the action of community representatives operating within a health service were identified in our analysis: 1) Building skills and confidence; 2) Engaging them in agendas for action: 3) Helping them navigate and understand the health system; and 4) Advocating to staff. A fifth role of advocating externally to outside groups and building coalitions is suggested as important, but was not strongly represented in our data.

**Conclusions:**

This study offers a new model synthesising the key roles of coordinating and facilitating community participation in health services which may be transferable to other health service settings. Our findings call attention to the need for health services to employ a facilitator who can support, engage, navigate and advocate for community representative’s participation and influence in health service policy and practice.

## Background

Prior to having a [Community Participation Coordinator] you wouldn’t get the chance to break out like we’re doing now and bring so many things in. (Community Representative, Interview)

While few people would argue against the principle of involving citizens in health system design and evaluation, in practice, the effectiveness of community participation has been questioned [1-4]. Most commonly recommendations to improve effectiveness of community participation have centered on training of community representatives^a^ (CReps) to understand the health system’s operations, and changing staff attitudes and organisational cultures to be more receptive to them [4-11]. Organisational characteristics argued as important in any partnership between health services and communities include: valuing the knowledge of community members [11,12]; facilitating access to information [12]; and developing staff skills in working with groups and in advocacy [13]. The idea of staff having advocacy and group development skills is also supported by the few studies which have included some investigation of the role of a salaried facilitator for community participation in health services [4,6].

### The role of a salaried facilitator in health services

A dedicated facilitator of community participation in health services has been identified as potentially important in improving engagement and influence of the community in service planning and delivery [4,6,10]. For instance, a salaried facilitator was argued as crucial to the influence of consumers in Cancer Partnership Projects in the United Kingdom for multiple reasons, including a key role in helping the group develop a shared vision based on “good interpersonal skills”. How the facilitator operated to support the consumers in their efforts was not elaborated [6]. We found no further studies which expanded on such a facilitator role, in particular how they may operate to improve CRep skills and knowledge and to influence health services and staff to be more receptive to the input and action of CReps. Patient navigators, who support individual patients accessing health services, are widespread in the United States and Canada [14] and have relevance here. A synthesis of patient navigator literature in cancer care found some evidence they increased timeliness of screening and treatment and identified their roles in helping patients overcome health system barriers, providing health education, addressing patient barriers to cancer care, and providing psychosocial support [14]. While the patient navigator role is more about supporting individual patients navigate the system as opposed to supporting CReps who are seeking to change the system more broadly, this literature provides a strong foundation for the value of a third party in health system navigation whether for individual care or advocacy for systems change.

In the absence of health service specific literature that details the role of a facilitator at the systems level, we turned to the literature at the nexus of community development and health promotion. Here, a central role is often advocated for an ‘empowering professional’ in supporting community members working to achieve structural changes in society, and community participation in decision-making sits centre stage [15-18]. How health professionals can support and empower a collective of community members to organise and effect change within a health system is potentially similar to how they may operate in a broader community context. The community development and health promotion fields are deeply informed by broader theories such as the socio-ecological model and open systems theories that highlight the need for structural change to support individual behaviour change [19]. The community development field makes strong claims for the role of a third party in facilitating community action. How this role is conceptualised by theorists and practitioners in these fields may inform understandings of the role of a facilitator of community participation in a health service context [15-18,20].

### Community development and the role of a third party

**Figure 1:**
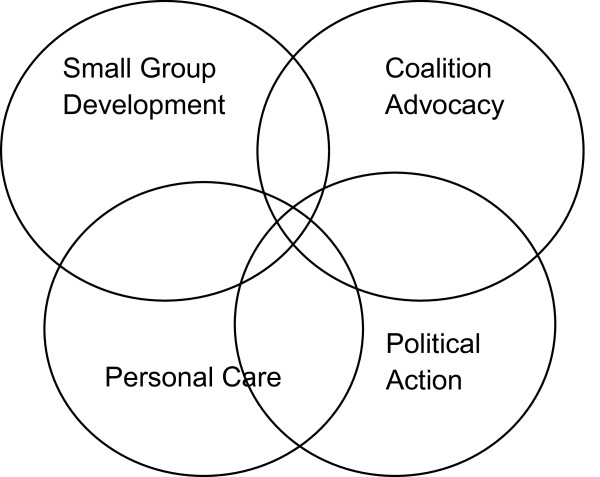
Labonte Empowerment Holosphere.

In reviewing the community development literature we found no consistent overarching framework or theory that helps researchers or practitioners to understand the role of a third party in enabling community members to influence decision-making and social change [15]. Despite strong claims for this third party role in community development practice [17,18], we found a lack of empirical research which systematically examined the role in practice. However, in the community development and health promotion field, we identified a widely cited approach to conceptualising this third party role which has been informed by practice and was developed from six years of training workshops with more than 2,500 community health practitioners in Canada [16]. This framework was considered potentially useful in understanding our study findings and is depicted in Figure 1.

Labonte sees the Empowerment Holosphere as a way of viewing professional practice as part of a strategy for social change in health promotion and community development and focuses on the professional’s role in this change process. The Holosphere is depicted with five spheres, each representing a different level of social organisation and relationship between a health professional, their organisation and the community. They overlap as one sphere can merge into the next with progression from the individual (personal care) to societal level (political action) suggested.

The **Personal care** sphere is focussed on personal empowerment of individuals and includes a professional dealing with individual needs and may align most closely with the Patient Navigator role [14]. **Group development**, the next sphere, is where a group of individuals forge an identity and create a purpose or agenda for action which is argued to enable participation in more structured processes or action for social change. This ‘mutual support’ phase is also a central element of other community development frameworks [15,18,21]. Community members turning their attention to issues beyond their immediate concerns are seen to occur as the group develops an identity of its own. The next sphere of **Community organisation** is the process of organising individuals around an agreed issue or problem which may be different to that of the professional’s organisation. The spheres of **Coalition building and Advocacy** are linked as one usually requires the other. The role of a health professional here is in aiding community groups by offering knowledge, analytical skills and an understanding of how the system or bureaucracy works and helping them join forces with groups with shared goals. The last sphere of **Political action** is an extension of advocacy and is more about a social movement where the group has political legitimacy and voice in the public arena. In the field of health promotion and community development, the professional is often working outside the agency that employs them, in the spaces of community groups and organisations in this sphere [18,22].

The case made for a third party in community development practice more broadly [15,18,21] and by Labonte specifically, predominantly focuses on what “the professional” should do to enable community organisation and action. How such a facilitator could operate in a health service setting, and what may restrain or facilitate action, is an important area of study. In health service settings involving the community and their ‘representatives’ is increasingly being supported, and an investigation of the role of a third party or facilitator in engaging the community in this context is timely [4,23,24].

### Aim

This paper examines the role of Community Participation (CP) Coordinators in facilitating community participation in the context of an Australian health service. The focus here is not on the impact on the community participants themselves. Other research has tackled this, finding that participation can have positive, empowering effects at the individual level [25,26]. Rather, this paper examines the kinds of actions and approaches taken by a staff member employed with the express purpose of coordinating, supporting and promoting participation, which may in turn facilitate the action and influence of CReps in a health service context.

## Methods

### Study design and setting

Data are drawn from an in-depth three year study of a Community Participation Program described elsewhere [11] involving multiple comparative case studies [27] in a large health district in Australia. Each case is one of eight Community Representative (CR) Networks operating at local hospitals in the health district. Two CR Networks and the hospitals in which they were located were focal points for data collection and were purposefully chosen as part of a larger study [28], and had well established CR Networks. The district has had community participation as a formal commitment since 2000 with an internally promulgated framework for community participation since 2004 [29]. The framework details the aims, structures, resources and training for community participation in the district. The aims are to 1) involve consumers, carers and the community in planning, delivery and evaluation of services; 2) keep local communities well informed; and 3) ensure there is transparency and accountability in decision-making and evaluation.

There are two main mechanisms for community participation in the district – local Community Networks at each hospital or acute health service which are open to all residents, and a CReps’ Council at the district level, in which each Network is represented by two elected members [11]. Part of the resources and structure to support community participation is a CP Coordinator employed at the local facility level reporting to the Hospital General Manager (GM). The key responsibilities of these positions outlined in the district level Framework are to:

1. Work closely with both staff and community to increase knowledge and skills in community participation;

2. Promote, recruit and support community representatives;

3. Advocate for and manage resources attached to community participation at the facility level;

4. Build capacity and provide on-going support for a culture of customer service.

### Ethics

The study was approved by University of New South Wales Human Research Ethics Committees (HREC 05081) and an Ethics Committee at the health service level (2006/012).

### Data collection

The focus of the study was on understanding the meeting points and interactions between distinct cultures, the health service workplace and CReps as a group, and was informed by an ethnographic stance [30-32]. Prolonged field engagement allowed informal and formal encounters to be included as data in fieldnotes and audio-recordings [32]. Sampling was purposeful with the choice of meetings and interview participants guided by the principles of seeking a diversity of views and meeting points, with emergent findings helping to direct further data collection [33,34]. A summary of the data collected is provided in Table 1. The data included multiple methods such as interviews, group discussions and observation across multiple sites over time with different stakeholders. This mixed methods approach allowed comparison of what was said by different stakeholders with what was observed, increasing confidence in the key findings presented about the role of the CP Coordinator [35].

**Table 1:** Summary of participants, interviews, observations, samples and purpose

**Participants**	**Number enrolled**	**Method**	**Data collected**	**Purpose**
CP Coordinators	Five staff, including one each from the two focal sites	In-depth, semi-structured interviews	Interview transcripts [8 hours, 10 minutes]	Views and perspectives of primary target group
CP Coordinators	As above plus two additional CP Coordinators	Group interview	Transcript [36 minutes]	Views and perspectives of primary target group
CReps	Ten key informant volunteers	In-depth, semi-structured interviews	Interview transcripts [11 hours 19 minutes]	Views and perspectives of secondary target group
Health service staff members	Nineteen purposively sampled clinicians and managers	In-depth, semi-structured interviews	Interview transcripts [15 hours 23 minutes]	Views and perspectives of secondary target group
Attendees at hospital committees	Members at ten meetings (10–15 members at each)	Non-participant observation	Recording & fieldnotes [10 hours, 58 minutes]	Interactions, issues discussion, resolutions, action points
Attendees at Community Network Meetings	Members at eleven meetings [6–10 CReps and 1–3 staff at each]	Non-participant and participant observations	Recording & fieldnotes [15 hours, 17 minutes]	Interactions, issues discussion, resolutions, action points
Attendees at CRep Council Meetings	Members at five meetings [15–20 CReps and 1-3staff at each]	Non-participant and participant observations	Recording & fieldnotes [9 hours, 12 minutes]	Interactions, issues discussion, resolutions, action points
Attendees at CP Coordinator Meetings	Members at four meetings [5–9 CP Coordinators at each]	Non-participant and participant observations	Recording & fieldnotes [6 hours, 55 minutes]	Interactions, issues discussion, resolutions, action points
Attendees at end of year, full day district Community Participation Conferences	Members at two meetings [Approx 70 attendees at each]	Non-participant observations	Recordings & fieldnotes [two full days of 14 hours combined]	Interactions, issues, presentations, discussion, resolutions, action points
**Totals**	**36 group and individual interviews, and over 30 CReps and 60 staff directly observed**		**56 hrs 22 mins: formal observation**	
			**36 hrs 18 mins: interviews**	

In-depth, semi-structured interviews were undertaken with ten CReps across the district who were considered key informants for the eight CR Networks and for the two focal sites in particular. Staff members at the two focal sites and key district managers were also interviewed (n=19). Formal interviews were also undertaken with five of the seven appointed CP Coordinators (one CP Coordinator covered two CR Networks) including the CP Coordinator at focal Site 1 and the staff member at focal Site 2 who was taking on this role in addition to his role in Patient Liaison, as well as three CP Coordinators from other sites. All CP Coordinators also discussed their role as a group in a meeting attended at the end of the second year of data collection. Each individual interview was 1–2 hours in length and explored participants’ espoused views about the role, expertise and influence of CReps and also the role of the CP Coordinator. They were audio-taped with consent and transcribed.

Data on enacted practice were collected via observations at a range of committees and meetings over three years up to 2010. The field researcher attended and recorded 10 health service meetings (10 hours 58 minutes) at the two focal hospital sites (such as clinical advisory and quality meetings), 11 Community Network Meetings (15 hours 17 minutes) at these same sites, five CRep Council Meetings (9 hours 12 minutes) and three CP Coordinator meetings (6 hours 55 minutes) at the district level as well as two end of year full day (7 hours each) district Community Participation Conferences in years two and three where all eight networks presented about their activities and achievements. This represented over 56 hours of formal ethnographic fieldwork in addition to interviews and time spent in the field over the three years interacting informally with staff and CReps. Fieldnotes were made during meetings, and conversations audiotaped with interactions and key events transcribed and then analysed [30]. Observation at health service meetings were non-participant whereas observations at Community Network, CReps’ Council and CP Coordinator meetings became more participant observations over time with the researcher being seen as a colleague sharing reflections from the research and from her own past experience as a CRep [36]. This approach was in keeping with an ethnographic stance where the researcher shifted between an Emic (insider) and Etic (outsider) perspective whilst in the field and during analysis [36]. The sharing of reflections between the researcher and participants assisted in the analysis and in the services ongoing development of community participation.

### Analytical approach

An iterative inductive approach to data analysis was taken. The analysis began in the first year of data collection with the critical role of the CP Coordinator highlighted early in the fieldwork. This finding guided subsequent data collection [33,34,37]. The data set was initially analysed thematically with constant comparison undertaken, that is, comparison of the views of different interviewees and comparison of what interviewees said with discussions and interactions which occurred in meetings [34,37,38]. NVIVO was used as a data management tool [39] with all data coded by the field researcher to nodes created and refined throughout the study. Further analysis for this paper focussed specifically on the node ‘Role of Coordinator’, which was coded to from all the data sets including Network Meetings at focal hospital Site 1 pre and post the appointment of a CP Coordinator. Site 2 did not have a dedicated CP Coordinator appointed during the study period. The CP Coordinator actions and roles articulated in interviews and observed during fieldwork were revisited and revised with constant movement between the different data sets and themes [34,38]. Comparison of the roles from this inductive analysis were then undertaken with the Empowerment Holosphere, which was used as a theoretical frame to inform the discussion of our findings [16].

## Results

In the presentation of our data, we will first illustrate the need for a CP Coordinator as a dominant theme in the community meetings attended early in the fieldwork. We will then turn to what happened after they were employed. Numbers are used to protect anonymity of the respondents yet also demonstrate the range of supporting data for our arguments. Salient observational data are captured from Site 1 where the discussions and actions of CReps can be examined pre and post appointment of a CP Coordinator. Actions of other Community Networks and their CP Coordinators, discussed in interviews, at district meetings and reported at the end of year Community Participation Conference, are also sources of data to illustrate the CP Coordinator role.

### “We need a coordinator”

The frustrations and impotence of CReps and their networks, operating without a CP Coordinator, were strongly reflected in early data collection. The problem of the employment of CP Coordinators was brought about by the amalgamation of health service areas into larger areas or districts just prior to the study which meant staff positions were lost, including CP Coordinators. The loss of CP Coordinators was acutely felt by many CReps as reflected in the following comments which were typical in early fieldwork.

Community representatives are planning on handing in their resignations. As you know we started off with nearly two people for so many different projects, we represented community and now what do we do, we’ve got nothing (CRep 1, Site 1 Network Meeting, Year 1).

Because we need a coordinator to actually recruit, sustain and you know do all these things to bring the group together (CRep2, Site 1 Network Meeting, Year 1).

Observations of Network Meetings at this time revealed a lack of direction and focus among community members. Very little proactive community participation was occurring with CReps mostly reporting back on committee activities and expressing frustrations at having no CP Coordinator. Calls for CP Coordinators were also common at the peak level CReps Council in observations in year 1 and the results of having no CP Coordinators were discussed at length, including people leaving the networks and no new members being recruited or supported:

We had a meeting on Friday – it was very badly attended. We need a coordinator (CRep3)

No hits, no runs, no coordinator (CRep4)

We need a coordinator (CRep5)

Advocacy by CReps for CP Coordinators to be appointed built over the following months with letters written and direct advocacy by long standing and well known CReps to service managers. By the end of the first year the battle to appoint CP Coordinators was reaping rewards *“We’re having some wins”* (CReps’ Council member) and by the end of year 2, there were four new dedicated CP Coordinators appointed including at Study Site 1.

### The core roles of the CP coordinator

**Figure 2:**
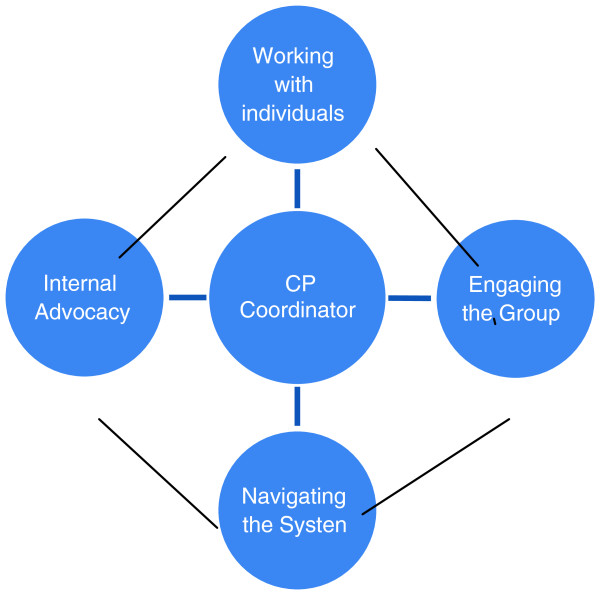
CP Coordinator roles.

The analysis of interviews and observations following the appointment of CP Coordinators led to the identification of four key roles for CP Coordinators as illustrated in Figure 2, and elaborated with supporting data below.

Four core CP Coordinator roles, that of working with individuals, engaging the group, navigating the system and advocacy, were identified during fieldwork. They are seen as inter-related and not necessarily occurring in a linear or ordered way. Each role could support the others, and entanglement of role behaviours was observed, illustrating the ongoing dynamics of CRep Networks and CP Coordinators support for individuals and the CR networks as a group. The connections between the roles are shown by the lines between the four spheres.

### Working with individuals

CP Coordinators spent some of their time working at the individual level with CReps; this was evident in both the interview and observational data. The importance of individual support and mentoring of CReps in their role in meetings and as advocates was often remarked upon in interviews with CP Coordinators and among CReps, as an important role of the CP Coordinator. CP Coordinators often commented on the need to empower individuals personally as well as collectively, as exemplified by the following extract:

People need time to be empowered, they need a sense of ‘we can do this’ and so that often it starts to shift if you start giving people a chance to actually perform and do well … It’s got to be kind of not actually be done for, but done with – and that’s sometimes just about done for, and then it’s done with and then it’s we’re doing (CP Coordinator 1, Interview).

The idea of gradually building skills and confidence in individuals by working alongside CReps was clearly evident in the way CP Coordinators describe their work and was observed in Network meetings and in ‘corridor conversations’ between the CP Coordinator and individual CReps at Site 1in particular. After the appointment of the CP Coordinator at Site 1, there were a number of instances of individual attention and support of network members by the CP Coordinator, for example, checking in on how they were doing as members of health service committees. The following is typical of the way the CP Coordinator at Site 1 regularly checked in with CReps:

CP Coordinator 3: Did you get to Infection Control? How did you go?

CRep1: Very very interesting yes [and then she went on to share her experience of the committee with the attentive focus of the CP Coordinator].

CP Coordinator 3: Are you still happy being part of that committee? Is it a productive committee?

CRep1: Yeah.

The value of just having someone to talk to was also a common theme among CReps when discussing the CP Coordinator role along with the mentoring and support CP Coordinators provided to individuals.

### Engaging the group

The data revealed a prominent role for CP Coordinators in engaging the group of CReps, primarily at Network meetings, with each other, with health staff and in discrete and achievable projects that were of interest and concern to the CReps. This role was further highlighted by the differences observed in the operation of the Network meetings at Site 1 before and after the CP Coordinator was appointed.

Both CReps and CP Coordinators across the health service talked about the role of the Coordinator in managing group dynamics, ‘bringing the group together’ - helping them to share their knowledge and experiences, and helping them mobilise around key issues. The comments below typify how study participants talked about this CP Coordinator role in working with the Network as a group:

I think a coordinator brings the group together [Lots of agreement with all talking at once]. You need that coordinator to hold everything together (CRep2 Site 1 Network Meeting, Year 3).

How can we work together as a group to mobilise this? How can we as a group share the knowledge and information that we have got together? I saw it more as a – my understanding of the network, like sharing information, looking at particular issues that we might be able to work together with (CP Coordinator 1, Interview).

Comparing the field researcher’s observations of Network Meetings at Site 1 before and after the appointment of the CP Coordinator it is evident that before their appointment CReps were attending committees and trying to stay engaged with the health service, but they were isolated and had little ability to progress their ideas and issues into any forms of concrete action. The group was also struggling to stay united (first quote), but after the CP Coordinator was appointed, Network meetings were observed as a place for ideas to be shared:

Well I dropped out because I got pissed off at somebody too. It’s taken a long time for me to turn around and come back. I’m back again because they said you were starting to move again (CRep3, Network Meeting Year 3).

The tone and dynamic of the meetings is just so different. People are more animated, excited and focussed on issues that matter to them (Field Notes, Year 2).

Later in the fieldwork CP Coordinators often spoke about the need to focus the group on small wins and discrete projects to get the CReps to feel engaged and work together. This issue was discussed at length at a CP Coordinator meeting in Year 2:

I think that they need to have wins so what I try to do is I have a huge horrible big plan, like transport, and then I have a small plan and so that we get a quick win and so we’re all interested and we stay engaged (CP Coordinator 2).

I think it’s really important to get them actually involved in projects so they can see some of the results of their work, rather than, they sit on committees … (CP Coordinator 3).

The CP Coordinator at Site 1 was also seen during the fieldwork to bring key health staff to Network Meetings to present about issues of interest and was a clear example of organising and engaging the Network around health service priorities and issues. It was also seen by CP Coordinators themselves as a way to involve CReps in health service activity beyond the usual practice of just appointing them to existing health service committees. The invitation and involvement of health staff became a common feature of the Network Meetings at Site 1 after the appointment of the CP Coordinator:

So at the next Network Meeting, I’m bringing the patient safety guy in, it’s a bit about letting the Reps know some of the things that we do within health, like in patient care. So I think it’s important to just build those connections, rather than always necessarily being on committees (CP Coordinator, Network Meeting, Site 1, Year 2).

When health staff attended Network Meetings, the CP Coordinator could be seen to actively facilitate the exchange between CReps and staff. The CReps were encouraged to ask the General Manager or staff member questions. CReps were also seen to work together at Network Meetings with staff and each other to think through solutions – something that wasn’t observed before the CP Coordinator was appointed. An example of this positive interaction between staff and CReps was seen in a discussion around patient safety at a Network Meeting:

Staff 1: So I’ve told you some of the contemporary adventures in patient safety.

CRep1: Well we’ve got so many questions and we’ve also got experiences.

[General Manager arrives]

GM: How are you all?

*CRep1****:****We’re having a very interesting discussion with [staff member’s name] he’s enlightening us. He’s explained some of the things we’ve had queries about.*

Staff 1: They dragged it out of me! [laughter around the table].

GM: Don’t let me interrupt that, just popping in to see how things are going.

CRep 1: I’ve recently had a nice holiday here at your expense. [laughter]. We were just telling [staff member’s name] a few of our observations and how to improve the system.

GM: Hope you wrote it all down [staff named followed by laughter around the table].

(Network Meeting, Site 1, Year 2)

In summary, the role of CP Coordinator in engaging group members with each other, health service staff and in small projects was reported as important in interviews and found to be critical to the action of CReps, particularly through the observations at Site 1. The lack of support for CReps for a period of time in the health service may have contributed to the prominence and value of this role at the time of the study as illustrated well by a CRep’s views about their lack of engagement and understanding of what was going on in the health service before CP Coordinators were appointed:

I feel there has got to be transparency and we don’t have that at the moment because we have no one representing us like the coordinator who can ask those questions and then they can pass it on to us (CRep9, Network Meeting Year 1).

The following roles of navigating the system and advocacy are presented separately to illustrate their different emphases. In practice, they often occurred simultaneously.

### Navigating the system

The CP Coordinator role of helping the CReps navigate the health system’s rules and procedures and understand how to make sense of health system information was reported in interviews and observed particularly at Site 1. The CP Coordinator appointed at Site 1 was seen frequently to offer the CReps knowledge, analytical skills and an understanding of how the health system works. These observations were supported by other CP Coordinators in interviews:

I met with the community members, we talked about what were the issues in the emergency department, we worked our way through the data, we actually identified key problems, we talked about how we would approach that (CP Coordinator1, Interview).

They need those, you know, basic skills in critical analysis. The ability to reframe those kinds of questions but also do their own sort of exploratory process to reach … you know a position where they can actually make an informed decision (CP Coordinator 4, Interview).

Examples of successful projects initiated and driven by CReps were the focus of presentations at the end of year Community Participation Conference in year 3. These projects were clearly facilitated by the CP Coordinator working with each of the Networks as the following excerpts from the end of year CP Conference illustrate. The CP Coordinator provided the understanding of how the bureaucracy worked and made sure the right people within the health service were consulted and involved to bring the CReps’ agenda into reality.

When I was sitting there one day and I thought why don’t we have a Ward Grannies out at [Hospital name] so I put it to [CP Coordinator named] and they just went what a great idea, let’s see what we can do. The Coordinator put it to [Hospital GM named] who said yep let’s do some studies. And some 18 months later, it took us a while to get off the ground … it wasn’t through lack of trying, it was all the paperwork and all the paraphernalia that goes behind it. I had no idea there was so much involved with being able to walk into a hospital and sit down and play with a child. The Ward Grandparents – we are mainly involved in the Paediatric Ward … and it’s just to give those Mums and Dads a break. (Network Member 3 presentation, Year 3)

Early this year (other rep named) and I had to spend time in the Cancer Therapy Day Treatment Centre. While we were sitting there we got to thinking the garden outside needed some Tender Loving Care. So we took the suggestion to our February reps meeting to look at the possibility of taking it on as a project … Our Coordinator put the wheels in motion and we got the go ahead from our GM and the Directors of Oncology … One of the reps took our idea to his Lions Club [a voluntary organisation] and Bunning’s [a large do-it-yourself hardware company] and after inspecting the site agreed they would help. Then things started cropping up … now the problems are solved and the work has started … planting is being done today. (Network Member 4 presentation, Year 3)

These cases illustrate clearly how the CP Coordinator accessed information, dealt with health system requirements and ensured staff support was in place for the projects to proceed. In contrast to the recognition of CP Coordinator’s key role by CReps, CP Coordinators often talked about the lack of prestige and recognition of their role among some health staff:

There is a lack of information about what we do and a lack of recognition of our role. They think you just sit in coffee shops! [Murmurs of agreement around the table] (CP Coordinator 2, Meeting, Year 2)

The role of the CP Coordinator in helping CReps navigate and understand the system, gain approvals and follow the correct procedures was reported as critical to the success of a number of community-sponsored projects which were established during the study period. The CP Coordinator however often went beyond simply assisting in navigating the system by directly advocating to internal decision-makers in the health service hierarchy for the community agenda.

### Advocacy

Advocacy is defined here as involving “the use of tools and activities that can draw attention to an issue, gain support for it, build consensus about it, and provide arguments that will sway decision makers and public opinion to back it” (p.2) [40]. The role of the CP Coordinator as an advocate for community projects was illustrated well in the examples of the Ward Grandparents and the Cancer Garden project presented earlier in which CReps highlighted the critical role of the advocacy of the CP Coordinator to key staff in the project’s success:

“*The advocacy of our Coordinator put the wheels in motion and we got the go ahead from our GM and the Directors of Oncology”. (Network Member presentation, Year 3)*

“The Coordinator put it to [Hospital GM named] who said yep”. (Network Member presentation, Year 3)

In addition to this role advocating the progression of specific CRep projects within the health service, CP Coordinators were also seen as advocates for community participation and for CReps in general terms, by staff and CP Coordinators in interviews:

And I think having the person standing at your door, reminding you and refreshing you that there’s a core responsibility that we’ve got … I think success is going to be very much driven, by the successes of the coordinators (Facility Manager, Site 1).

I guess that’s part of my role – is about educating some of the staff that people are not just tokenistic (CP Coordinator 3, Interview).

Advocacy was seen however, by a number of interviewees, as a delicate balancing act as CP Coordinators sit between the health service and the community – accountable to both:

Being able to work outside the hospital to kind of get messages out there as well as working within the four walls … I think you’ve got to work both. Somehow you’ve got to balance the needs of both … you’re accountable to both but it’s not undoable (CP Manager, Interview).

Both staff and CReps commented that CP Coordinators promoted the value of community participation in the community more broadly. However, forging links between CRep networks and external organisations on specific agendas for action and change was not observed or reported during fieldwork nor suggested by CReps or Coordinators as a key approach to progressing an issue. Engaging external groups in health services processes was also at an early stage when the study completed. For example, though there was talk among some CP Coordinators of setting up Multicultural Access Committees and liaison with Aboriginal communities as examples of building links with external partners this was not realised during the study period. There was only one mention in the interview data of the role of CP Coordinators in building external relationships or partnerships although it was a focus of discussion at a later CP Coordinator’s meeting in relation to bringing these external groups into the health service arena as participants in their processes:

Work around Aboriginal liaising is a focus at the moment, having the Aboriginal liaison person out to look at the facility (CP Coordinator 2, Meeting, Year 2).

## Discussion

The aim of this article was to identify and articulate key roles and actions of staff members whose purpose it is to support and promote participation of the community in health service decision-making. In our study the CP Coordinator role appeared central to CReps becoming a more cohesive group and pursuing their own community-focussed agenda over time in the health service sites where they were employed. The four core roles we identified for CP Coordinators were not discharged in any linear or hierarchical progression as suggested in Labonte’s Holosphere [16]. Instead, these ways of acting in support of CReps were found to be utilised by CP Coordinators as needed to exploit opportunities as they presented themselves, for example facilitating the contribution of CReps at a Network Meeting at Site 1 to new developments in improving patient safety.

At sites where a designated CP Coordinator was appointed in year 2 of the study, the CReps reported harnessing the relationships and knowledge of the Coordinator to achieve their own ends, as evidenced in the Cancer Garden and the Ward Grandparents Program examples. It may be the case that CReps can identify opportunities, but a CP Coordinator is important in navigating the rules and procedures of the health service and to advocate to key staff to effect change.

Our findings and approach to understanding the role of CP Coordinators in facilitating participation provides a new model, and more detailed and nuanced insights into how such a facilitator of community participation may operate in a health service context than previous studies [4,6,10,41]. Our data illustrate the role of the CP Coordinator assisting CReps to achieve their aims by working with the group opportunistically, engaging with staff and helping CReps navigate the health system, in essence, to exploit the diverse web of power relations [42,43] in a health service to achieve the CReps’ agenda for change.

The key roles of the CP Coordinator in the domains of engaging the group and navigating the system can be seen to align with Labonte’s group development and community organisation spheres of action [16]. Here the CP Coordinator helps the group forge a collective identity and purpose and assists in organising them around common problems, in addition to promoting their participation in existing processes within the system. Previous studies in community participation in health services point to the importance of moving beyond group development to directly influence wider system decision-making, in short, to avoid consumers simply becoming a support group for each other [4,6,41]. Labonte goes further to argue that professionals working with community groups need to consider carefully what they are encouraging them to participate in – a bureaucratic, superficial process or a process where real change can occur [16]. Our data reveal that the CReps at the health service studied did move beyond sharing experiences as a group in a potentially tokenistic and isolated process of exchange to making changes to practices, environments and policies of the health service with the support of the CP Coordinator. We also saw CP Coordinators actively questioning the kinds of projects and activities that CReps were encouraged to participate in and fostering projects where they could “see the results of their work”.

Nonetheless questions remain about the extent of the role of the CP Coordinator, for example, as an advocate, and how their advocacy may be constrained within health service settings. The health service is continuing to examine the CP Coordinator role and how it can be enhanced. We have also published further work from the research which details the action and effects of CReps in the health services we studied, with and without the assistance of a CP Coordinator [44]. The advocacy role of the CP Coordinator in our study was found to be focussed on helping the CReps navigate the system they are in, access resources and gain the right approvals to proceed with their activities. Advocacy and coalition building, including promoting CReps’ agendas to outside groups as conceptualised by Labonte’s Empowerment Holosphere, were actions largely absent in the data we collected. Coalitions are argued as central to the effectiveness of community action in the broader community development field [16,18] and their potential utility to CP Coordinators working within a health service to achieve change should not be discounted. The ability of CP Coordinators to actively and overtly encourage coalitions with groups operating outside of the health service requires further investigation. Such external advocacy and coalition building is suggested as a possible strategy as soon as the group begins to engage in health service issues and develop their own priorities or agendas for action. It may be the case that the length of time that the CP Coordinator role was observed was not sufficient to see them move from internal relationship-building to the external realm.

CP Coordinators did not express any frustrations about their mostly internal focus though they wanted to engage more with external groups and sometimes perceived their role as misunderstood and not always valued by other staff. Lack of understanding among staff may represent a challenge in the CP Coordinators’ quest to meet the agenda of the health service as well as pursuing community identified interests and projects, as a lack of staff support may be an obstacle to changes to policy and practice. A key role of the CP Coordinator in the framework studied is to work closely with staff as well as the community to improve knowledge and skills to work together and our findings suggest this should encompass promotion of their own role. The current lack of recognition of the CP Coordinator’s role among some health staff suggested by our findings could translate to a lack power for them in their own health organisation, restricting their ability to work with CReps on mostly smaller projects and to those within the confines of the health service.

## Conclusions

This study found four key roles for the CP Coordinator which facilitate the activities of CReps: providing support to individuals as representatives and advocates; engaging them as a group by providing knowledge of what is going on in the health service, supporting identification of issues for action and a response; helping CReps navigate the system to progress their own agendas; and advocating the value of community participation and CReps’ agendas within the heath service. The role of the CP Coordinator in external advocacy and coalition building is suggested as important, but was not evidenced during the time of the study.

The actions and roles of CP Coordinators identified in this study contribute to knowledge about how a health service staff member employed to support and promote community participation can operate to enhance CReps’ engagement and contribution to improving health services for patients. Our analysis offers new understandings of a third party or facilitator role, working with the community in the specific setting of a health service, which may be transferable to other similar health service settings. The roles we identified build on and elaborate conceptual tools in community development practice and identify potential limitations of this facilitator role in the context of a health service. We recommend commitments to fostering meaningful community participation in health services should include the employment of a facilitator who can undertake the roles we found to be central to CReps’ engagement and participation in the study sites. Active recognition and encouragement of the facilitator roles we have identified are needed among health staff and managers if community participation in health services is to make lasting improvements to the patient experience.

## Endnotes

^a^CReps is used as an all encompassing term for patient, carer, consumer or community representatives

## Competing interests

The authors declare that they have no competing interests.

## Authors’ contributions

SN designed the study, conducted all fieldwork and undertook primary analysis. All authors participated in the refinement of the qualitative data analysis and presentation in the paper. SN drafted the paper and JB and NS commented on drafts with SN leading the revision process. All authors have read, commented and approved the final manuscript.

## Pre-publication history

The pre-publication history for this paper can be accessed here:

http://www.biomedcentral.com/1472-6963/13/154/prepub

## References

[B1] Contandriopoulos D A sociological perspective on public participation in health care Soc Sci Med 2004 58 2 321 330 10.1016/S0277-9536(03)00164-3 14604618

[B2] Learmonth M Martin GP Warwick P Ordinary and effective: the Catch-22 in managing the public voice in health care? Health Expect 2009 12 1 106 115 10.1111/j.1369-7625.2008.00529.x 19250156 PMC5060472

[B3] Martin GP Representativeness, legitimacy and power in public involvement in health-service management Soc Sci Med 2008 67 11 1757 1765 10.1016/j.socscimed.2008.09.024 18922611

[B4] Attree P Morris S Payne S Vaughan S Hinder S Exploring the influence of service user involvement on health and social care services for cancer Health Expect 2011 14 1 48 58 10.1111/j.1369-7625.2010.00620.x 20673242 PMC5060558

[B5] Daykin N Evans D Petsoulas C Sayers A Evaluating the impact of patient and public involvement initiatives on UK health services: A systematic review Evid Policy 2007 3 1 47 65 10.1332/174426407779702201

[B6] Sitzia J Cotterell P Richardson A Interprofessional collaboration with service users in the development of cancer services: The Cancer Partnership Project J Interprof Care 2006 20 1 60 74 10.1080/13561820500515304 16581640

[B7] Stewart S Watson S Montague R Stevenson C Set up to fail? Consumer participation in the mental health service system Australas Psychiatry 2008 16 5 348 353 10.1080/10398560802047367 18608147

[B8] McCann TV Baird J Clark E Lu S Mental health professionals' attitudes towards consumer participation in inpatient units J Psychiatr Ment Health Nurs 2008 15 1 10 16 10.1111/j.1365-2850.2007.01199.x 18186824

[B9] Gagliardi AR Lemieux-Charles L Brown AD Sullivan T Goel V Barriers to patient involvement in health service planning and evaluation: An exploratory study Patient Educ Couns 2008 70 2 234 241 10.1016/j.pec.2007.09.009 18023129

[B10] Draper M Involving consumers in improving hospital care: lessons from Australian hospitals Commonwealth Department of Health and Family Services 1997 Canberra, Australia:

[B11] Nathan S Johnston L Braithwaite J The role of community representatives on health service committees: staff expectations vs. reality Health Expect 2011 14 3 272 284 10.1111/j.1369-7625.2010.00628.x 21029280 PMC5060580

[B12] Putland C Baum F MacDougall C How can health bureaucracies consult effectively about their policies and practices? Some lessons from an Australian study Health Promot Int 1997 12 4 299 309 10.1093/heapro/12.4.299

[B13] Germann K Wilson D Organizational capacity for community development in regional health authorities: a conceptual model Health Promot Int 2004 19 3 289 298 10.1093/heapro/dah303 15306613

[B14] Wells KJ Battaglia TA Dudley DJ Garcia R Greene A Calhoun E Mandelblatt JS Paskett ED Raich PC Patient Navigation Research P: **Patient navigation: State of the art or is it science** Cancer 2008 113 8 1999 2010 10.1002/cncr.23815 18780320 PMC2679696

[B15] Hur MH Empowerment in terms of theoretical perspectives: Exploring a typology of the process and components across disciplines J Community Psychol 2006 34 5 523 540 10.1002/jcop.20113

[B16] Labonte R Health promotion and empowerment: reflections on professional practice Health Educ Q 1994 21 2 253 268 10.1177/109019819402100209 8021151

[B17] Toomey AH Empowerment and disempowerment in community development practice: eight roles practitioners play Community Dev J 2011 46 2 181 195 10.1093/cdj/bsp060

[B18] Minkler M Community Organising and Community Building for Health and Welfare 2012 3 New Brunswick: Rutgers University Press

[B19] Archer S Kelly C Bisch S Systems and holism (Chapter 1) in Implementing change in communities: a collaborative process 1984 St Louis: Mosby

[B20] Labonte R Victoria RL **Power and empowerment: Building transformative relations from the inside out** Power, Participation and Partnerships for Health Promotion 1997 Australia: Victorian Health Promotion Foundation 27 41

[B21] Jackson T Mitchell S Wright M The Community Development Continuum Community Health Stud 1989 13 1 66 73 2736907 10.1111/j.1753-6405.1989.tb00178.x

[B22] Baum F The New Public Health 2008 3 Melbourne: Oxford University Press

[B23] Australian Council on Healthcare Standards National Report on Health Services Accreditation 2007–2008 2009 Sydney: Australian Council on Healthcare Standards

[B24] Leonhardt KK HRET patient safety leadership fellowship: the role of "community" in patient safety Am J Med Qual 2010 25 3 192 196 10.1177/1062860609357469 20354232

[B25] Christens BD Peterson NA Speer PW Community Participation and Psychological Empowerment Health Educ Behav 2011 38 4 339 347 10.1177/1090198110372880 21460177

[B26] Wilson PM Kendall S Brooks F The Expert Patients Programme: a paradox of patient empowerment and medical dominance Health Soc Care Community 2007 15 5 426 438 10.1111/j.1365-2524.2007.00701.x 17685988

[B27] Stake R Denzin N, Lincoln Y Case Studies Handbook of Qualitative Research 2000 2 California: Sage 435 454

[B28] Braithwaite J Greenfield D Westbrook J Pawsey M Westbrook M Gibberd R Naylor J Nathan S Robinson M Runciman B Health service accreditation as a predictor of clinical and organisational performance: a blinded, random, stratified study Qual Saf Health Care 2010 19 1 14 21 10.1136/qshc.2009.033928 20172877

[B29] Consumer and Community Participation Framework http://www.sswahs.nsw.gov.au/sswahs/community/pdf/CP_Framework.pdf

[B30] Denzin N Lincoln Y Handbook of Qualitative Research: 2nd Edition 2000 California: Sage

[B31] Parker R Ehrhardt A Through an Ethnographic Lens: Ethnographic Methods, Comparative Analysis, and HIV/AIDS Research AIDS Behav 2001 5 2 105 114 10.1023/A:1011399426632

[B32] de Laine M Ethnography: Theory and Applications in Health Research 1997 Sydney: MacLennan and Petty

[B33] Patton M Qualitative Research & Evaluation Methods 2002 London: Sage Publications

[B34] Charmaz K Constructing grounded theory: A practical guide through qualitative analysis 2006 London: Sage

[B35] Bazeley P Kemp L Mosaics, Triangles, and DNA: Metaphors for Integrated Analysis in Mixed Methods Research J Mixed Methods Res 2012 6 1 55 72 10.1177/1558689811419514

[B36] Spradley JP Participant Observation 1980 New York: Wadsworth, Thomson Learning

[B37] Hesse-Biber SN Leavy P The Practice of Qualitative Research 2011 2 California: Sage Publications

[B38] Braun V Clarke V Using thematic analysis in psychology Qual Res Psychol 2006 3 77 101 10.1191/1478088706qp063oa

[B39] QSR International NVIVO 9 2011

[B40] Rice M Advocacy and Reproductive Health – Success and Challenges Promot Educ 1999 VI 2 3

[B41] Brooks F Nursing and public participation in health: An ethnographic study of a patient council Int J Nurs Stud 2008 45 1 3 13 10.1016/j.ijnurstu.2006.08.012 17046769

[B42] Foucault M The Foucault reader 1986 Harmondsworth: Penguin

[B43] Foucault M The history of sexuality. Vol. 3: The care of the self 1990 London: Penguin

[B44] Nathan S Stephenson N Braithwaite J Side-stepping questions of legitimacy: How community representatives manoeuvre to effect change in a health service Health Interdiscip J Social Study Health, Illness Medicine Published online before print January 30, 2013, doi: 10.1177/1363459312473617). http://www.ncbi.nlm.nih.gov/pubmed/23364312

